# Omega-3 Eicosapentaenoic Acid Decreases CD133 Colon Cancer Stem-Like Cell Marker Expression While Increasing Sensitivity to Chemotherapy

**DOI:** 10.1371/journal.pone.0069760

**Published:** 2013-07-16

**Authors:** Flavia De Carlo, Theodore R. Witte, W. Elaine Hardman, Pier Paolo Claudio

**Affiliations:** 1 Department of Biochemistry and Microbiology, Joan Edwards School of Medicine, Marshall University, Huntington, West Virginia, United States of America; 2 McKown Translational Genomic Research Institute, Joan Edwards School of Medicine Marshall University, Huntington, West Virginia, United States of America; 3 Department of Surgery, Joan Edwards School of Medicine, Marshall University, Huntington, West Virginia, United States of America; University of Navarra, Spain

## Abstract

Colorectal cancer is the third leading cause of cancer-related death in the western world. *In vitro* and *in vivo* experiments showed that omega-3 polyunsaturated fatty acids (n-3 PUFAs) can attenuate the proliferation of cancer cells, including colon cancer, and increase the efficacy of various anticancer drugs. However, these studies address the effects of n-3 PUFAs on the bulk of the tumor cells and not on the undifferentiated colon cancer stem-like cells (CSLCs) that are responsible for tumor formation and maintenance. CSLCs have also been linked to the acquisition of chemotherapy resistance and to tumor relapse. Colon CSLCs have been immunophenotyped using several antibodies against cellular markers including CD133, CD44, EpCAM, and ALDH. Anti-CD133 has been used to isolate a population of colon cancer cells that retains stem cells properties (CSLCs) from both established cell lines and primary cell cultures. We demonstrated that the n-3 PUFA, eicosapentaenoic acid (EPA), was actively incorporated into the membrane lipids of COLO 320 DM cells. 25 uM EPA decreased the cell number of the overall population of cancer cells, but not of the CD133 (+) CSLCs. Also, we observed that EPA induced down-regulation of CD133 expression and up-regulation of colonic epithelium differentiation markers, Cytokeratin 20 (CK20) and Mucin 2 (MUC2). Finally, we demonstrated that EPA increased the sensitivity of COLO 320 DM cells (total population) to both standard-of-care chemotherapies (5-Fluorouracil and oxaliplatin), whereas EPA increased the sensitivity of the CD133 (+) CSLCs to only 5-Fluorouracil.

## Introduction

Colorectal cancer is the third leading cause of death from cancer in the western world and each year is responsible for a half million deaths worldwide [1], [2]. Despite receiving surgical resection and chemotherapy, nearly 50% of patients develop resistance, tumor relapse, or metastatic diseases [2], [3]. Recent discoveries have shown that this may be due, at least in part, to the existence of cancer stem-like cells [4] and this highlights the need for improved therapies that can target them.

A growing body of evidence lends support to the idea that human cancer can be considered a stem cell disease. The cancer stem cells model proposes that only a fraction of cells within a tumor possess cancer initiating potential and that these cancer stem-like cells (CSLCs) are able to initiate and sustain tumor growth [5], [6]. Ricci-Vitiani [4] and O'Brian [7] were the first to provide independent proof of the existence of colon CSLCs. They isolated a CD133 (+) population of cells within the tumor that was capable of growing as undifferentiated colon-spheres in a serum-free media, which could be differentiated into the heterogeneous tumor cell population. They demonstrated that only the CD133 (+) subpopulation was tumorigenic in a serial xenograft assay in immunodeficient NOD/SCID mice. Recently, it has been shown that CD133 may also be used to identify CSLCs in established cell lines. CD133 (+) cells isolated from cancer cell lines have been found to be more tumorigenic than the CD133 (−) cellular fraction both *in vitro* and *in vivo* [8]. Additionally, CD133 (+) cells, grown in spheres, maintain the expression of this marker even in long-term sphere culture [9]. Recent evidence from clinical studies showed that CD133 expression was negatively correlated with patient prognosis in advanced colon cancer [10], [11].

Epidemiologic studies have shown an increased incidence of colon cancer in populations consuming a western diet, rich in red meat, animal fat, and low in grains. This incidence is reduced in subjects that consume higher amounts of fruits, vegetables, fibers, and, more importantly, n-3 polyunsaturated fatty acids (PUFAs) of marine origin [12], [13]. The parent n-3 PUFA α-linolenic acid (ALA) is found in vegetable oil, while its metabolites eicosapentaenoic acid (EPA) and decosahexaenoic acid (DHA) are obtained predominantly from cold-water fatty fish. Much data have been published about the capability of n-3 PUFAs to decrease proliferation, to exert a pro-apoptotic effect, and to inhibit angiogenesis in several *in vitro* models of colon cancer [14]–[17]. Omega-3 PUFAs have also been shown to increase the sensitivity to chemotherapy of several human derived cancer cell cultures *in vitro* such as breast [18], brain and lung [19], lymphocytic [20], and colonic [15]. In a similar way, n-3 PUFAs sensitized *in vivo* models of breast cancer [21], sarcoma [22], and leukemia [23] to anticancer therapy. However, all of these studies addressed the effects of PUFAs treatments on the bulk of tumor cells, not on the CSLCs.

The antitumor activity of n-3 PUFAs has not only been linked to their effects on proliferation and apoptosis but also on differentiation in different cell models. A link between omega-3 PUFAs and promotion of cellular differentiation has already been described in normal and malignant cells [24]–[28]. Omega-3 PUFAs have been shown to differentiate myeloid progenitor cells in the bone marrow of mice without altering the number of white blood cells in circulation [24]. In a similar way, it has been shown that long term treatments of EPA and DHA, which does not change the morphology or the average numbers of cells in the crypt section of normal colonic mucosa in rat, did reduce cellular proliferation, enhance differentiation and apoptosis [29].

Additionally, treatment of human breast cancer cells with n-3 PUFAs resulted in their growth inhibition, which was proportional to mammary gland differentiation [27], and a pro-differentiating effect was also observed in DHA treated human melanoma cells *in vitro* [26].

The purpose of our study was to investigate if *in vitro* concentrations of EPA equal to plasma levels achievable in the human body following supplementation of the diet with 2.4 g n-3/day [30], were able to affect the differentiation status and chemosensitivity of the overall population of colon cancer cells and specifically of the CD133 (+) CLSCs.

## Materials and Methods

### Cell culture

The human colorectal adenocarcinoma cell line COLO 320 DM (CCL-220, Duke's C) was obtained from the America Type Culture Collection (Rockville, MA). Colo320DM cells were cultured in RPMI-1640 supplemented with 10% fetal bovine serum, 2 mM L-glutamine, Penicillin 100 U/mL and Streptomycin 100 µg/mL (HyClone, South Logan, UT). Cells were subcultured at a density of 1×10^5^/ml twice a week in a humidified atmosphere at 37°C, 5% CO_2_.

### Chemicals and drugs

Eicosapentaenoic acid, EPA (Omega-3 polyunsaturated fatty acid, 20∶5) was purchased from Cayman Chemicals (Ann Arbor, MI) and stearic acid (SA, saturated fatty acid, 18∶0) from Sigma-Aldrich (St. Louis, MO). 100 mM stock solutions of EPA, and SA in absolute ethanol were made and diluted to final concentration with culture media.

Oxaliplatin and 5-Fluorouracil were purchased by Alexis Biochemicals (Farmingdale, NY). 12.6 mM stock solutions of Oxaliplatin and 384.3 mM 5-Fluorouracil in DMSO were made and diluted to final concentration with culture media.

Control cells were treated with the same amount of vehicle, ethanol or DMSO (CTRL VH). The final vehicle concentrations never exceeded 0.1% v/v.

### Gas Chromatography

COLO 320 DM were plated in 100 mm petri dishes (1×10^6^ cells) and treated after 4 hours with vehicle control or a range of concentrations of EPA and SA (6.25 uM, 12.5 uM, 25 uM). The fatty acid compositions of COLO 320 DM were assessed using gas chromatography. CTRL VH and treated cells were harvested after 96 hours, washed in PBS1X to remove surface lipids, then homogenized in distilled water with 0.1% BHT (3,5-di-*tert*-butyl-4-hydroxy-toluene) in methanol to prevent fatty acid oxidation. Lipids were extracted with chloroform/methanol, and then transmethylated. Methylated lipids were separated and identified using gas chromatography, as previously published [31]. Fatty acid methyl ester standards (Nu-Chek-Prep, Elysian, MN) were used for peak identification.

The individual fatty acid methyl esters of EPA, DPA (decosapentaenoic acid), DHA and, SA were reported as the percent of the sum of the total methylated fatty acids (area under the curve).

### Metabolic activity assay (MTT assay) time course

COLO 320 DM cells were plated in 96-well plates (3,000 cells/well). After 4 hours, cells were treated with a range of concentrations of EPA and SA (6.25 uM, 12.5 uM, 25 uM) or vehicle control (CTRL VH) for 0–4 days. Cells were then analyzed daily for metabolic activity by MTT assay. Briefly, 10 ul of 5 mg/ml MTT (3-(4, 5-Dimethylthiazol-2-yl)-2, 5-diphenyltetrazolium bromide) (Sigma-Aldrich, St. Louis, MO) were added to each well. The plates were incubated for 2 hours in a humidified atmosphere at 37°C, 5% CO_2_. After this time media was removed, the formazan salt formed was solubilized with DMSO and the absorbance was read at 570 nm with a micro-plate reader.

### Cell count and Fluorescence-activated cell sorting (FACS) analysis

COLO 320 DM were plated in a 24-well plate (2.5×10^4^/well) and treated after 4 hours with a range of concentrations of EPA or SA (6.25 uM, 12.5 uM, 25 uM) for 96 hours. Viable cells were counted using a trypan blue exclusion method. Subsequently cells were suspended in cold FACS buffer (PBS1X, 2 mM EDTA, 0.1% BSA) and aliquots of cells, 2×10^5^ cells/50 ul, were labeled with the appropriate concentration of monoclonal antibody against CD133/2 (293C3), phycoerytrin conjugated (Miltenyi Biotech, Bergisch Gladbach, Germany). An isotype antibody was used as a control antibody for background normalization. Analysis was carried out with an Accuri-C6 Flow Cytometer (BD Accuri Cytometers, Ann Arbor, MI). After the percentage of positivity to CD133 was determined, the number of CD133 (+) and CD133 (−) negative cells within the total population was calculated.

### Real Time PCR

COLO 320 DM were plated in 100 mm petri dishes (1×10^6^ cells) and treated with vehicle, EPA, or SA (25 uM). Cells were harvested after 48 and 96 hours and total RNA was extracted from the samples using Trizol according to the manufacturer's protocol (Invitrogen Life Technologies, Carlsbad, CA). RNA was solubilized in ddH2O and nucleic acid concentration was measured by a Nano Drop 2000 (ThermoFisher Scientific, Waltham, MA). RNA (1 ug) was subjected to reverse-polymerase chain reaction using a High Capacity cDNA transcription Kit (Applied Biosystems, Foster City, CA) and Real-time PCR was performed with RT2 SYBR Green/ROX qPCR Master Mix using an ABI PRISM 7000 Sequence Detection System (Applied Biosystems, Foster City, CA). Relative changes in gene expression were calculated using the 2-ΔΔCt method, described by Livak and Schmittgen [32]. β-Actin was used as housekeeping gene.

β*-Actin-F*: GGT TCC GCT GCC CTG AGG;

β*-Actin-R*: GTC CAC GTC ACA CTT CAT G.

Specific pairs of primers were used to amplify genes of interest:

*CD133-F*: TTG GCT CAG ACT GGT AAA TCC C;

*CD133-R*: ATA GGA AGG ACT CGT TGC TGG T;

*CK20-F*: ACC TCC CAG GCC TTG AGA T;

*CK20-R*: TGG CTA ACT GGC TGC TGT AA;

*MUC2-F*: GGG ACT GTG AGT GCT TCT GC;

*MUC2-R*: AGT GGA TGC CGT TGA TGG T.

### Western Blotting

COLO 320 DM were plated in 100 mm petri dishes (1×10^6^ cells) and treated with vehicle, EPA or SA (25 uM) for 48 and 96 hours. Total proteins were extracted from cells after lysis in cold sample buffer (150 mM NaCl, 50 mM Tris/HCl, pH 8, 0.5 mM EDTA, 0.1 mM EGTA, 1% Triton X-100). Sodium dodecylsulphate–polyacrylamide gel electrophoresis (SDS–PAGE) was carried out according to the Laemmli method [33]. Equal amounts of total proteins of each sample were subjected to electrophoresis then transferred to 0.45 um nitrocellulose membranes. After blocking in 5% fat-free-milk in TBS-T, membranes were incubated with the following antibodies: Anti-CD133/1 (AC133 Miltenyi Biotech, Bergisch Gladbach, Germany), Anti-Cytokeratin 20 (EPR1622Y Abcam, Cambridge, MA). Anti-β Actin (Sigma-Aldrich, St. Louis, MO) was used as loading control. Following the incubation with the horseradish peroxidase-conjugated secondary antibodies, the immunocomplexes were visualized by enhanced chemiluminescence detection system (Amersham Biosciences, Piscataway, NJ). Images were acquired using the Luminary/FX imaging system (Fotodyne, Hartland, WI). Densitometry was performed with Image j 1.45 software (Image Processing and Analysis in Java) (imagej.nih.gov/ij/download/).

### Dot Blotting

Dot blot was performed as previously described [34]. Briefly, COLO 320 DM were plated, treated with vehicle, EPA or SA (25 uM) for 48 and 96 hours and proteins were extracted as described in the western blot analysis section. Equal amounts of total proteins of each sample were spotted on a 0.45 um nitrocellulose membrane. A peptide competition assays was performed to verify the specificity of the Muc-2 antibody (Pierce, ThermoScientifics, Waltham, MA). 300 µg of custom made peptide (CPDFDPPRQENETWW, peptide 2.0, Chantilly, VA) with an identical sequence to the one used to generate the Muc-2 antibody was incubated for 2 hours at room temperature with 1 µg of muc-2 antibody. After blocking in 5% fat-free-milk in TBS-T, membranes were incubated with anti-Muc-2 and anti-β Actin (Sigma-Aldrich, St. Louis, MO) as a control. Following the incubation with the horseradish peroxidase-conjugated secondary antibodies, the immunocomplexes were visualized and imaged as described in the western blot analysis section.

### Isolation of Cancer Stem Cell population

CSCLs were isolated using a CD133 Microbead kit (Miltenyi Biotech, Bergisch Gladbach, Germany). COLO 320 DM total cell population was labeled with a monoclonal antibody CD133/2 (293C3) conjugated to micro-beads and the CD133 (+) cells were magnetically sorted. An aliquot of cells eluted from the column was labeled with a monoclonal antibody against CD133/1, phycoerytrin conjugated (AC133, Miltenyi Biotech, Bergisch Gladbach, Germany) and a FACS analysis was performed to evaluate the percentage of purity of the CD133 (+) cells.

### Growth-inhibition assay

COLO 320 DM cells were plated in 96-well plates (3,000 cells/well). After 4 hours, cells were treated for 24 hours with a range of concentrations of oxaliplatin (0.005–0.1 mM) or 5-Fluorouracil (0.05–2 mM) to determine the concentration causing 25% and 50% of growth inhibition. Cells were then analyzed for their metabolic activity by an MTT assay.

### Chemosensitivity assay

COLO 320 DM (3×10^3^) cells/wells, total population or CD133 (+), were plated in 96-well plates. After 4 hours, complete media supplemented with vehicle, EPA, or SA was added to achieve a final concentration of 25 uM FAs. After 48 hours the media was replaced with fresh media supplemented with a range of oxaliplatin (0.005–0.1 mM) or 5-Fluorouracil (0.05–2 mM) concentrations. After 24 hours of treatment an MTT assay was performed to define the inhibitory concentration that lead to a reduction in viability of 25% (IC25) and 50% (IC50).

### Statistical analysis

All experiments were carried out at least three times and presented as means ± SD. All data were analyzed using Prism© software (Graphpad, Inc., La Jolla, CA). One-way analysis of variance (ANOVA) with Tukey multiple comparisons post-test was used to determine statistical significance of the differences between experimental groups: *p* values less than 0.05 were considered statistically significant.

## Results

### Eicosapentaenoic Acid is incorporated and metabolized by COLO 320 DM cells

**Figure 1:**
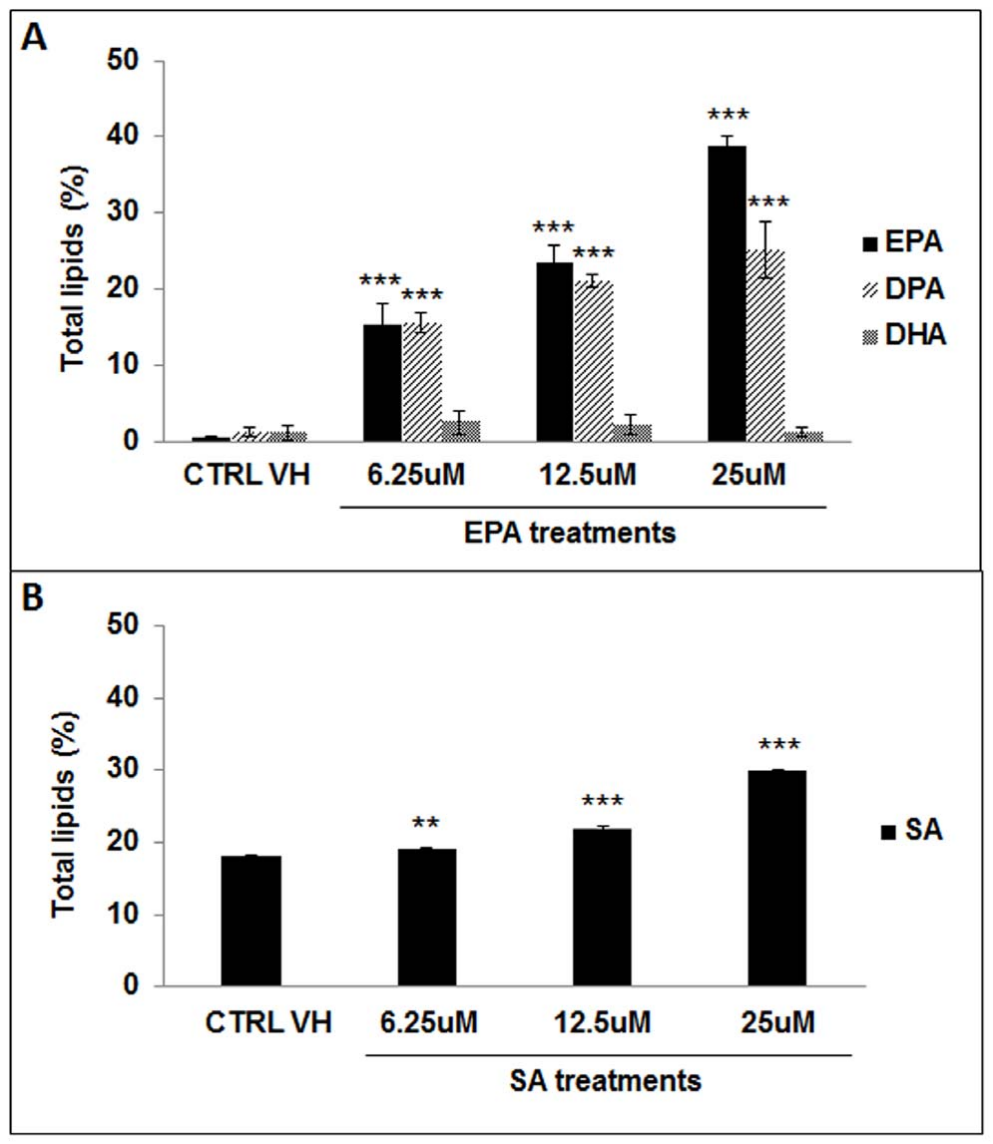
Analysis of fatty acids incorporation by gas chromatography. Total lipids were extracted from COLO 320 DM cells treated for 96 hours with control vehicle (CTRL VH) or a range of concentrations of (**A**) EPA or (**B**) SA, (6.25–12.5–25 uM). Gas chromatography was performed to study the fatty acids incorporation and their metabolites by COLO 320 DM cells. Results are presented as relative percentage with respect to the CTRL VH. Results represent the mean ± SD of at least three experiments. *Black bars*: compound used for the treatment detected with GC; *Light Grey bars*: DPA (*docosapentaenoic Acid*, C22:5, n-3); *Dark Grey bars: DHA (docosahexaenoic Acid, C22:6, n-3)*. (SA: stearic acid C18:0).

To evaluate the incorporation of fatty acids by COLO 320 DM cells we analyzed the fatty acids composition by gas chromatography after treatment with EtOH (CTRL VH) or with a range of concentrations (6.25 uM, 12.5 uM, 25 uM) of PUFAs n-3, EPA, or saturated FAs SA. After 96 h the cells were harvested and the total lipids were extracted. First we detected that SA is 30-fold more represented in the control vehicle total lipid cellular membrane than EPA, DPA, and DHA. We observed (**figure 1A**) a significant dose responsive increase of EPA in the cells treated with this fatty acid. Moreover the fraction corresponding to DPA, which is an EPA metabolite, also increased after EPA treatment. These results showed that COLO 320 DM cells incorporated and efficiently metabolized EPA. In a similar way we observed a dose dependent increase of SA content in the total lipids from COLO 320 DM treated with stearic acid (**figure 1B**).

### 25 uM Eicosapentaenoic Acid treatment decreased the cell number of COLO 320 DM cells without affecting the CSLCs population

Several lines of evidence have shown the existence of a small population of cancer stem-like cells that can initiate and maintain tumors. The use of specific markers, including CD133 for colon CSLCs, has allowed researchers to label these cells and distinguish between the CSLCs population and the bulk of tumor [4], [7]. Omega 3 fatty acids have already been reported to reduce the bulk of tumor cells [14]–[17], but the effect on CSLCs has not been determined.

**Figure 2:**
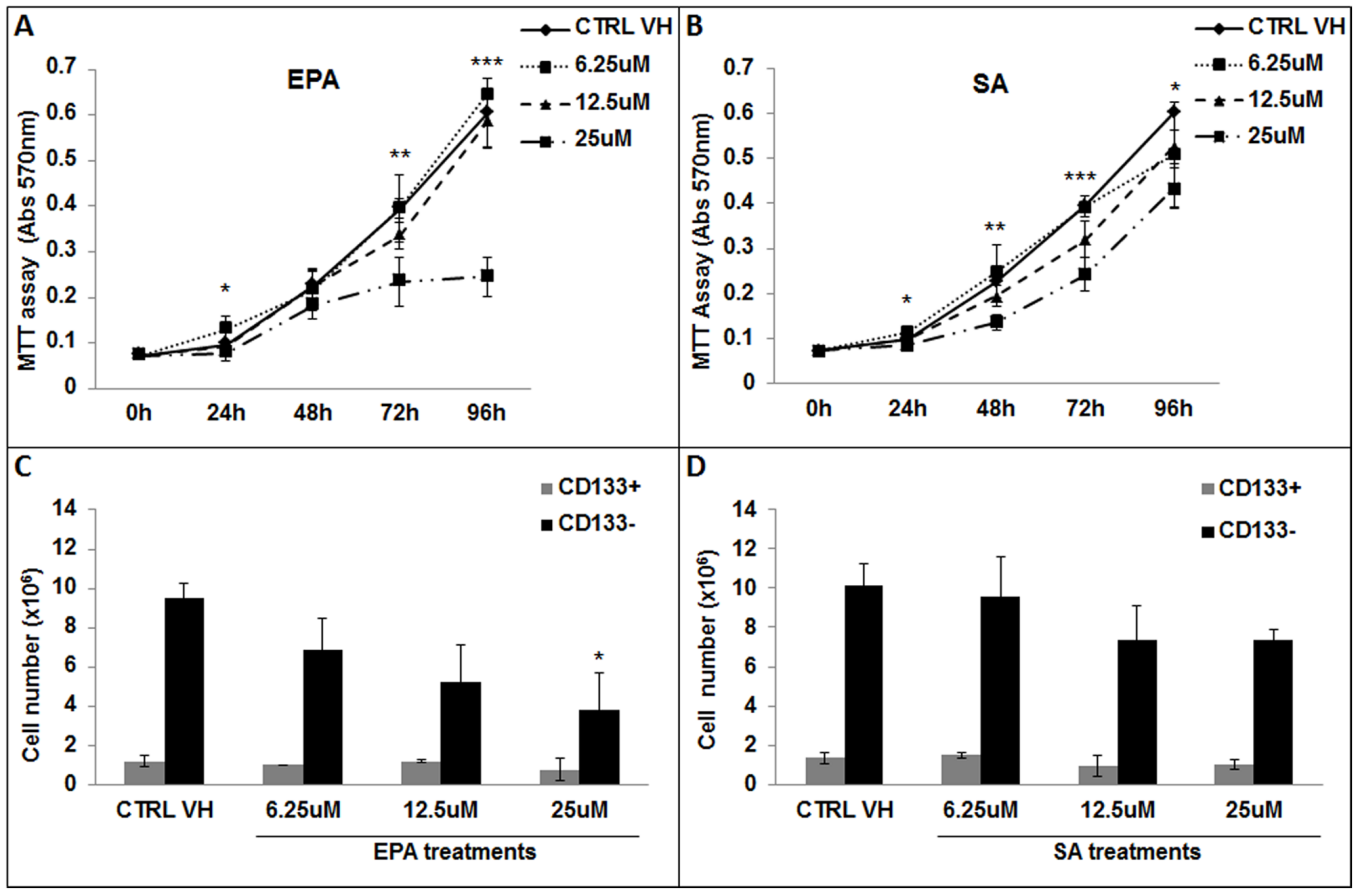
Differential effects of fatty acids on COLO 320 DM CD133 (+) and CD133 (−) cell number. COLO 320 DM cells were treated for 0–96 hours with vehicle control (CTRL VH) or 6.25–12.5–25 uM of (**A**) EPA and (**B**) SA. MTT assay of EPA and SA treated cells vs. vehicle control (CTRL VH) treated cells. *p* values were calculated with one-way ANOVA test with a Tukey's multiple comparison post-test on the various treatments within each time point. *p≤0.05; **p≤0.01; ***p≤0.001; n = 5. COLO 320 DM cells were also treated for 96 hours with vehicle control (CTRL VH) or a range of concentrations of fatty acids (6.25–12.5–25 uM), (**C**) EPA and (**D**) SA. Viable cells were counted and labeled with a monoclonal antibody anti-CD133 to determine the percentage of CD133 (+) cells in the treated groups. Results represent CD133 (+) and CD133 (−) cell numbers of ± SD of at least three experiments. *p* values were calculated with Student's t-test on treated samples vs. CTRL VH (* p≤0.05).

**Figure 2A** and **Table 1** show that a time course treatment (0–96 hours) with 25 uM EPA significantly decreased the number of COLO 320 DM (total population) as measured by MTT assay with respect to the vehicle control (p<0.01 at 72 hours; and p<0.001 at 96 hours). Treatments with 25 uM SA reduced to a less extent the cell number of the COLO 320 DM cells (**Figure 2B** **and** **Table 1**).

**Table 1:** Tukey's Multiple Comparison Test on Figure 2.

EPA								
	24 h		48 h		72 h		96 h	
	Significance	p value	Significance	p value	Significance	p value	Significance	p value
**CTRL VH vs. 6.25 uM**	ns		ns		ns		ns	
**CTRL VH vs. 12.5 uM**	ns		ns		ns		ns	
**CTRL VH vs. 25 uM**	ns		ns		**	≤0.01	***	≤0.001
**6.25 uM vs. 12.5 uM**	ns		ns		ns		ns	
**6.25 uM vs. 25 uM**	*	≤0.05	ns		**	≤0.01	***	≤0.001
**12.5 uM vs. 25 uM**	ns		ns		ns		***	≤0.001

SA								
	24 h		48 h		72 h		96 h	
	Significance	p value	Significance	p value	Significance	p value	Significance	p value
**CTRL VH vs. 6.25 uM**	ns		ns		ns		ns	
**CTRL VH vs. 12.5 uM**	ns		ns		*	≤0.05	ns	
**CTRLVH vs. 25 uM**	ns		*	≤0.05	***	≤0.001	*	≤0.05
**6.25 uM vs. 12.5 uM**	ns		ns		*	≤0.05	ns	
**6.25 uM vs. 25 uM**	*	≤0.05	**	≤0.01	***	≤0.001	ns	
**12.5 uM vs. 25 uM**	ns		ns		*	≤0.05	ns	

*p≤0.05;

**p≤0.01;

***p≤0.001; n = 5.

In order to define which cellular population (bulk of tumor or CSLC) was affected, we first treated COLO 320 DM with a range of concentrations of EPA or SA (6.25 uM, 12.5 uM, 25 uM) for 96 hours. EPA differently affected the two cellular sub-populations (CD133 (+) and CD133 (−) cells). A trypan blue cell count was performed after we labeled aliquots of treated and untreated cells with a monoclonal antibody against CD133/2 (glycosylated epitope 2). A fluorescence-activated cell sorting analysis was performed to determine the percentage of cells positive to CD133 and to calculate the number of CD133 (+) cells in the total population for each treated sample. **Figure 2C** shows that treatments with EPA significantly decreased (p<0.05) the number of CD133 (−) cells (bulk of tumor), at the physiologic concentration of 25 uM. Interestingly, the CD133 (+) CSLCs population was only barely affected by the same concentration of EPA (25 uM) that caused a reduction in cell number of the CD133 (−) sub-population. The treatment with SA, which is a saturated fatty acid, did not significantly change either CD133 (+) or CD133 (−) cell number (**figure 2D**). In conclusion, we showed that 25 uM EPA significantly reduced the number of COLO 320 DM bulk of tumor cells, but that SA did not.

### Eicosapentaenoic Acid induced an increase in colonic epithelium differentiation markers, Cytokeratin 20 and Mucin 2, mRNA expression while decreasing CSLCs marker, CD133

The intermediate filament Cytokeratin 20 is expressed in epithelial cells of the gastrointestinal tract. CK20 has been shown to be downregulated or its expression is completely lost in a number of colorectal cancer patient samples when compared to the adjacent normal mucosa [35]–[37]. Similarly, MUC2 is less expressed in colorectal adenocarcinoma, while its expression is maintained in normal tissue adjacent to the malignancy [38]–[40].

**Figure 3:**
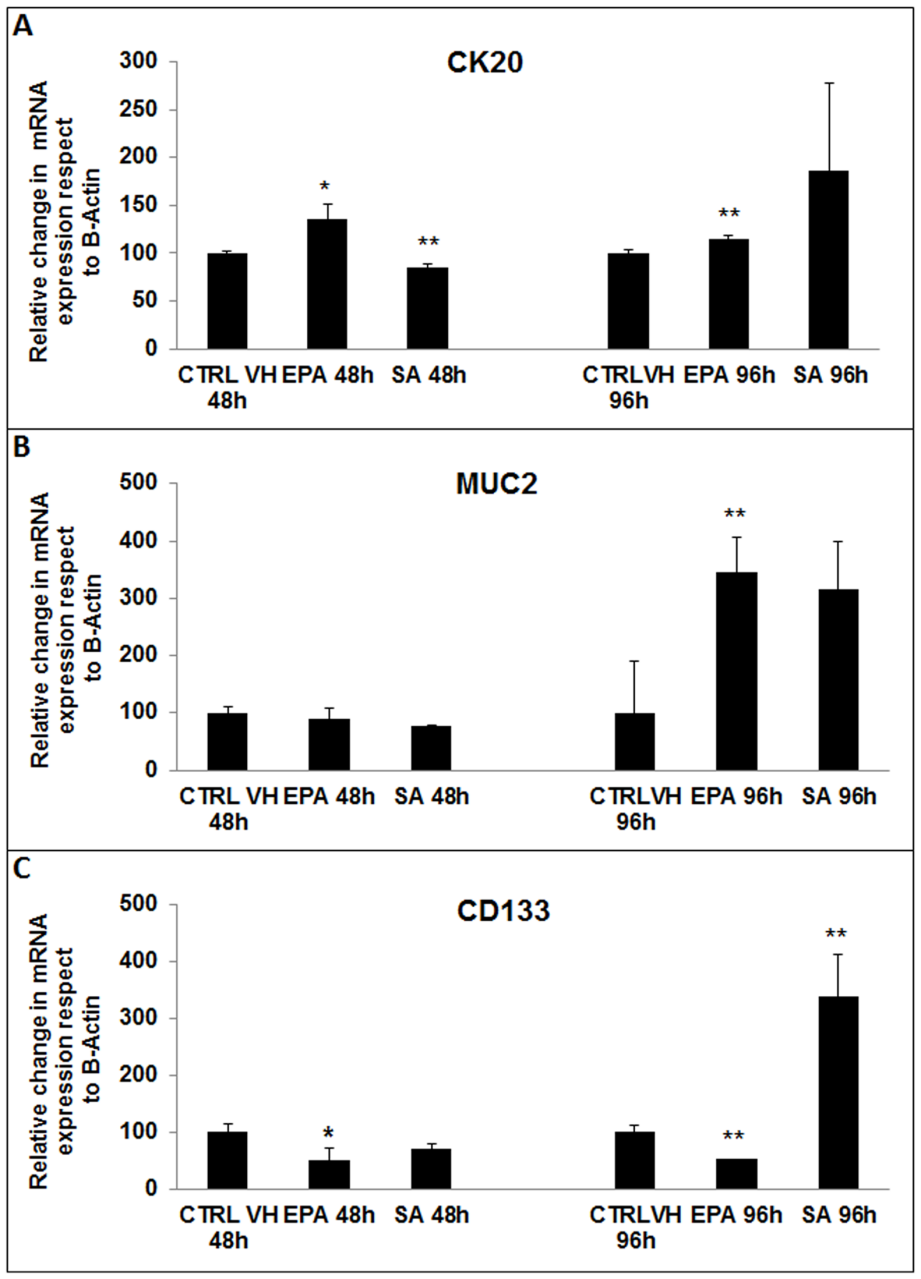
Real-Time PCR of colon differentiation and CSLCs markers after fatty acids treatments. COLO 320 DM cells were treated with vehicle control (CTRL VH) and 25 uM of EPA or SA for 48 and 96 hours. Total RNA was extracted and Real-Time RT-PCR was performed to study the relative change in gene expression of specific markers when compared to β-Actin. The expression of the differentiation markers (**A**) cytokeratin-20 (CK20), an enterocytes and goblet cells marker, (**B**) Mucin-2 (MUC2) a goblet cells marker, and (**C**) colon cancer stem-like cells marker CD133 were studied after treatments with vehicle control (CTRL VH), EPA or SA. Results represent the mean ± SD of at least three experiments. *p* values were calculated with Student's t-test on treated samples vs. CTRL VH (* p≤0.05, ** p≤0.01).

CD133 has been reported to specifically mark the small undifferentiated population of tumor initiating cells in colon cancer [4], [7] and to be associated with poor prognosis in advanced colon cancer [10], [11]. To understand if EPA altered gene expression of CK20, MUC2, and CD133, we treated COLO 320 DM for 48 and 96 hours with 25 uM EPA, SA, or vehicle control. We observed that EPA induced an increase in the relative mRNA levels of CK20, found on both enterocyte and goblet cells (**figure 3A**), and of the goblet cells marker MUC2 (**figure 3B**), compared to the vehicle treated cells. The observed effects were statistically significant at both 48 and 96 hours for CK20 and at 96 hours for MUC2. We also observed that EPA induced a decrease in the expression of the CD133 CSLCs marker at 48 and 96 hours (**figure 3C**) (p<0.05 and 0.01). SA (**figure 3A, 3B**) significantly decreased CK20 at 48 hours of treatment without affecting MUC2 expression. We also observed that SA, on the other hand, increased CD133 RNA expression (**figure 3C**) at 96 hours following treatment. This data suggests that omega-3 has a pro-differentiation effect on COLO 320 DM cells.

### 25 uM Eicosapentaenoic Acid increases Cytokeratin 20 protein synthesis while decreasing CD133 in COLO320 DM cells

**Figure 4:**
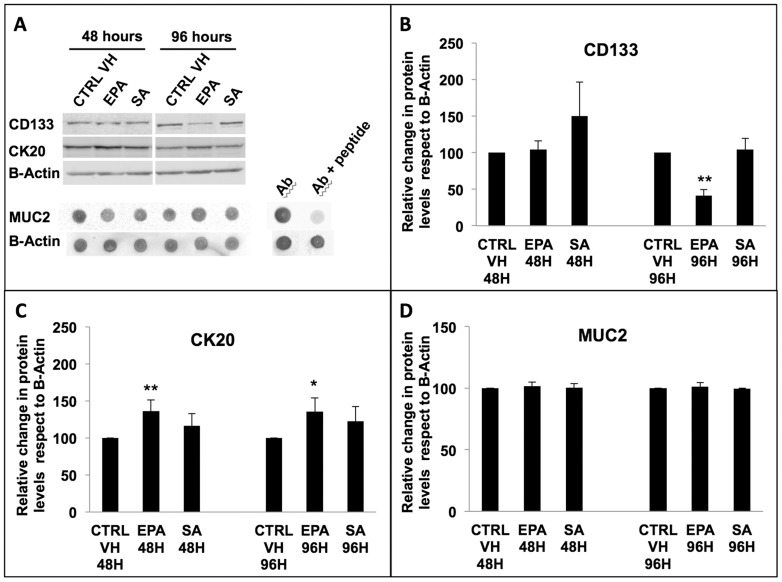
Western blot and dot blot analysis of colon differentiation and CSLCs markers following fatty acids treatments. (**A**) Total lysate were obtained from COLO 320 DM cells treated with vehicle control (CTRL VH), 25 uM EPA or SA for 48 and 96 hours. Western blot was performed to study the expression of cytokeratin-20 (CK20) and CD133 after treatment with the fatty acids. Dot blot was performed to study the expression of Mucin 2 (MUC2) after treatment with the fatty acids. A peptide identical to the one used to generate the Muc-2 antibody was used in a peptide competition assay to determine MUC2 antibody specificity. The changes in (**B**) CD133, (**C**) CK20, and (**D**) MUC2 protein expression with respect to β-Actin are representative of three separate experiments. Results represent the mean ± SD of at least three experiments. *p* values were calculated with Student's t-test on treated samples vs. CTRL VH (* p≤0.05, ** p≤0.01).

To determine if the change in mRNA expression correlated to an effect on CK20, Mucin 2, and CD133 protein synthesis, we extracted the total protein content from COLO 320 DM following treatment for 48 and 96 hours with vehicle, EPA and SA at 25 uM. As predicted by Real Time PCR analysis, the CD133 protein was significantly decreased after 96 hours of EPA treatment (**figure 4A and B**). We observed that at 48 and 96 hours Cytokeratin 20 protein levels were significantly higher in 25 uM EPA treated cells when compared to control vehicle or to the SA treatment (**figure 4A and C**) as demonstrated by western blot analysis. We also observed that Mucin 2 protein levels did not change at 48 and 96 hours by dot blot analysis in 25 uM EPA treated cells when compared to control vehicle or to the SA treatment (**Figure 4A and D**). To verify the specificity of the muc-2 antibody that was used for dot blot analysis, we conducted a peptide competition assay and have shown that 300 fold excess of peptide efficiently competed the binding of the antibody to the muc-2 present in the cell lysate.

In conclusion, we observed that EPA increased the protein expression of the marker of differentiation CK20, and decreased the expression of the marker of stemness CD133 in COLO 320 DM cells.

### Pre-treatment with 25 uM Eicosapentaenoic Acid increases the sensitivity of COLO 320 DM total population and CSLCs to 5-Fluorouracil

The ability of omega-3 fatty acids to improve the efficacy of anticancer drugs both *in vitro* and *in vivo* on bulk of tumor cells has been previously reported [15], [18], [19], [22], [23], [31]. However, still unreported are the chemo-sensitizing effects of EPA on the bulk of tumor compared to the effects on the CSLCs population following standard-of-care anticancer treatments.

In this work we investigated whether pretreatment with EPA increases the chemotherapy response of standard-of-care anticancer drugs in both bulk of tumor cells and CSLCs population cultured from COLO 320 DM cells.

**Figure 5:**
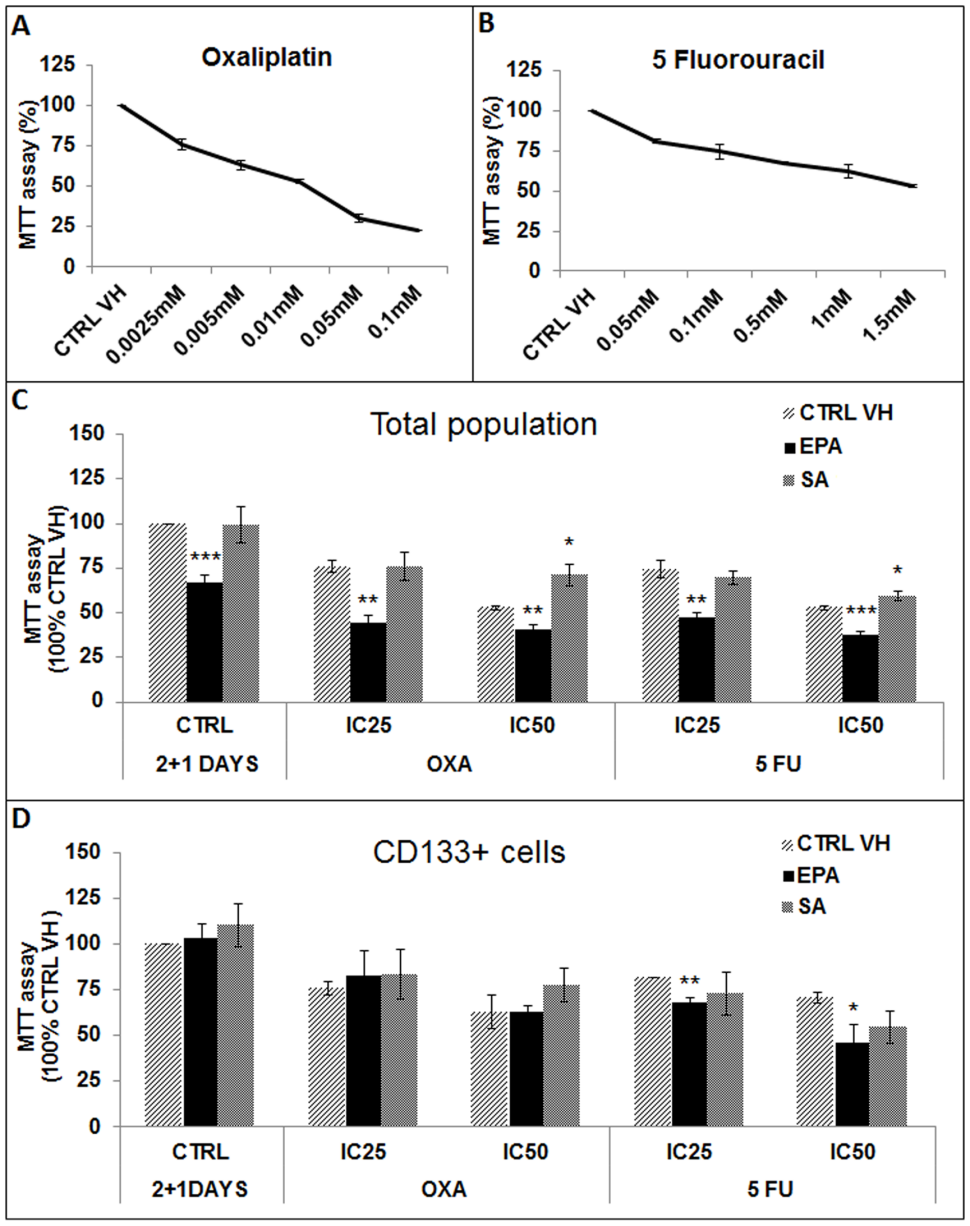
Sensitivity of COLO 320 DM total population and CSLCs cells to Oxaliplatin and 5-Fluorouracil following treatment with 25 uM EPA. (**A**) COLO 320 DM cells were treated with a range of Oxaliplatin (0.005–0.1 mM) or 5-Fluorouracil (0.05–2 mM) concentrations to determine the inhibitory concentration of 25% (IC25) and 50% (IC50). (**B**) Cells from COLO 320 DM total population were pre-treated for 48 hours with 25 uM EPA or SA. Afterwards cells were exposed for 24 hours with Oxaliplatin (*IC25, 2.5* *uM; IC50, 10* *uM*) and 5 Fluorouracil (*IC25, 100* *uM; IC50, 1.5* *mM*). (**C**) CD133 (+) cells were magnetically sorted from the total population of COLO 320 DM and were pre-treated for 48 hours with 25 uM EPA or SA and then exposed for 24 hours to IC25 and IC50 of Oxaliplatin and 5-Fluorouracil. Results represent the mean ± SD of at least three experiments. *p* values were calculated with Student's t-test on treated samples vs. CTRL VH (* p≤0.05, ** p≤0.01, *** p≤0.001).

We found that 2.5 uM or 10 uM oxaliplatin resulted in a cell viability of 75.88±3.23% (IC_25_) or 53±1.18% (IC_50_) of control (**figure 5A**). We found that 100 uM and 1.5 mM 5-Fluorouracil decreased COLO 320 DM cell viability to 74.48±4.85% (IC_25_) and 52.90±1.09% (IC_50_) of control (**figure 5B**).

To determine if n-3 PUFA was able to increase the sensitivity of the total population of COLO 320 DM cells to chemotherapy, we plated and pre-treated the cells as before with 25 uM of the EPA or SA fatty acid. After 48 hours we removed the media and treated the cells for 24 hours with the previously defined IC25 and IC50 concentrations for oxaliplatin and 5-Fluorouracil (**Figure 5** **A and B**). We found that EPA, but not SA (**figure 5C**, light gray bars), significantly improved the efficacy of both chemotherapeutic drugs (**figure 5C**, black bars), with respect to the control vehicle.

To define if the same treatment with 25 uM of EPA fatty acid similarly affected CLSCs response to chemotherapy, we tested the chemosensitivity of magnetically sorted CD133 (+) COLO 320 DM cells to oxaliplatin and 5-Fluorouracil. CD133 (+) CSLCs were pre-treated with 25 uM EPA for 48 hours, which was followed by a treatment for 24 hours with oxaliplatin or 5-Fluorouracil. As previously demonstrated by others [41], we observed that CD133 (+) CSLCs were more resistant to the antineoplastic drugs tested (**figure 5D**, dark gray bars) when compared to the total population (**figure 5C**, dark gray bars). Interestingly, we found that EPA pre-treatment (black bar), but not SA (light grey bar) significantly increased the sensitivity of CD133 (+) CSLCs to 5-Fluorouracil, but not to oxaliplatin (**figure 5D**).

Collectively these data suggest that EPA potentiates the efficacy of 5-Fluorouracil, which is one of the first line standard-of-care antineoplastic agents, against colon cancer.

## Discussion

There is a growing focus on diet and the use of naturally abundant compounds as supplements because they have many beneficial properties on human health with minimal side effects. Our study indicates that n-3 consumption (fish) instead of stearic acid consumption (red meat) may decrease the viability and proliferation of CSLCs.

Polyunsaturated fatty acids of the n-3 series (ALA, EPA, DPA docosapentaenoic acid, and DHA) are considered essential fatty acids because eukaryotic cells cannot make the omega-3 bond and it must be provided in the diet. The saturated stearic acid (SA) and its n-9 derivatives can be acquired from both endogenous biosynthesis and dietary intake.

COLO 320 DM is a Duke's C derived colorectal adenocarcinoma cell line that was used in this work to study stage III, non-metastatic cancer. Many studies have previously focused on only one cell line to study the effects of fatty acids as well as other compounds [42]–[50].

We did not study locally advanced colon cancer cell type because surgery alone is their standard-of-care (stage 0–2 colorectal cancer, Duke's A, B). Surgery and adjuvant chemotherapy are instead standard-of-care for stage 3 colorectal cancer (Duke's C). Various combinations of neaoadjuvant chemotherapy, adjuvant chemotherapy, surgery and radiation therapy are the standard-of-care for Duke's D colorectal cancer. Therefore, this research excluded cell lines classified as Duke's A, B and metastatic Duke's D colorectal carcinomas. Understanding cellular signaling pathways that regulate colon CSLCs differentiation along with the determination of simple dietary interventions aimed at differentiating both bulk of tumor cells, and CSLCs, may results in the establishment of adjuvant therapies with the aim to increase the sensitivity of locally advanced colon cancer to standard-of-care therapy.

In our first experiment we wanted to determine the uptake of EPA and SA, by gas chromatography. We observed that in COLO 320 DM control vehicle, the total lipid cellular membrane content of EPA, DPA, and DHA was 30-fold lower than the content of SA. After treating the cells for 96 hours with a range of concentrations of fatty acids, we detected that the intake of EPA was dose dependent; the cells actively incorporated and metabolized EPA to DPA. Modification of DPA to DHA was not observed as shown previously by others [29]. On the other hand, the incorporation of SA, although dose dependent, was modest and this could be explained by the fact that SA is already well represented in the cell membranes of the COLO 320 DM cells.

Omega-3 fatty acids have already been reported to decrease malignant cell growth, but their effects were only studied on the bulk of tumor cells [14]–[17]. Evidence has been published about the existence of a small subset of cancer stem-like cells that can initiate and maintain tumors. Antibodies reacting against specific markers, such as CD133 for colon CSLCs, can be used to label the cancer stem-like cells population inside the tumor [4], [7]. As previously observed by others, we showed by MTT assay that 25 uM EPA significantly reduced the cell number of COLO 320 DM total population after 72 and 96 hours. However, our main interest was to determine if the n-3 fatty acid EPA would cause unique effects on the CSLCs when compared to the bulk of tumor cells. After a 96 hours treatment with EPA or SA, using a specific antibody against CD133, we individually analyzed the effects of the fatty acids treatments on the CD133 (+) colon CSLCs, and CD133 (−) cells. We observed that EPA treatments, in comparison to SA, induced a dose dependent reduction of cell number that was specific to the CD133 (−) sub-population, reaching a significant effect at 25 uM (p<0.05). On the other hand, we did not observe changes in cellular number in the CD133 (+) CSLCs treated with either EPA or SA. Interestingly, a small decrease in CD133 (+) CSLCs number, even if not statistically significant, was detected after treatment of the cells at 25 uM EPA.

PUFAs of the n-3 series have been shown to promote cellular differentiation of the myeloid progenitors in the hematopoietic system, cells of mammalian glands, pre-adipocytes, human breast cancer and melanoma cells [24]–[28]. In order to define if cellular differentiation was one of the processes induced by EPA treatment in the COLO 320 DM cells, we studied the trend of expression of specific differentiation markers for the colonic epithelium and colon cancer stem-like cells. It has already been shown in CaCo2 and HT29 cell lines that the induction of differentiation by Sodium Butyrate can reduce the expression of the CSLCs markers CD133 and CD44 [51]. In a similar way, cultures of HT116 cells in three-dimensional colon-spheres show increased expression of differentiation markers, CK20 and MUC2, when the cells are induced to differentiate [52].

We observed that a treatment with 25 uM EPA for 48 hours up-regulated CK20 and down-regulated CD133 mRNA expression. The same treatment induced after 96 hours an up-regulation of both CK20 and MUC2 and down-regulation of CD133 mRNA expression levels.

Western blotting and dot blotting were used to verify these data by showing an increase in CK20 protein expression at both 48 and 96 hours following EPA treatment and a decrease of CD133 expression at 96 hours. Although we showed a statistically significant increase in the mRNA levels of Mucin 2 at 96 hours, we did not observe a significant change in Mucin 2 protein expression either at 48 or 96 hours. This indicates that times longer than 96 hours could be required to detect Mucin 2 mRNA translation into protein.

These observations could indicate that EPA may induce a more differentiated status of the bulk of tumor cells, and that on the other side it may trigger the reduction of the stemness status of the CSLCs, as evidenced by a reduction of the CD133 marker expression.

The SA-induced decrease in mRNA expression of CK20 at 48 hours and the up-regulation of CD133 expression at 96 hours could be linked to the previously demonstrated pro-inflammatory properties of the stearic acid [53]. However, the SA-induced effects on CK20 and CD133 mRNA expression were not supported by corresponding changes in protein expression leading to the hypothesis that this could be due to a regulation only at the mRNA level.

Our observation that EPA specifically triggers the transition of the CSLCs to a more differentiated subset led us to the idea that omega-3 treatments could increase the sensitivity of the CSLCs to standard-of-care chemotherapy agents. The beneficial effects of omega-3 treatments in increasing the sensitivity to chemotherapy of bulk of tumor cells *in vitro* and *in vivo* [15], [18]–[23] and in reducing its side effects [54] have been described. However, all past studies addressed the effects of omega-3 fatty acids treatments on the bulk of tumor cells, not on the CSLCs.

The standard adjuvant chemotherapies for stage-III colon cancer are 5-Fluorouracil plus leucovorin or oxaliplatin/leucovorin/5-Fluorouracil [55]. Traditional platinum derivatives have generally been ineffective in colorectal cancer therapy; however, the third-generation platinum derivative oxaliplatin has shown good antitumor activity and a lack of cross-reactivity with cisplatin [56]. In general, CD133 (+) cells and serum free culture of small spherical cells has been found less sensitive to oxaliplatin and 5-Fluorouracil than CD133 (−) cells [41]. In the present study we sought to determine if the omega-3 fatty acid EPA, which we found to increase the differentiation of COLO 320 DM cells, could sensitize CD133 (+) CSLCs to standard-of care chemotherapies.

We showed that the efficacy of oxaliplatin or 5-Fluorouracil mono-therapies on the COLO 320 DM total population was considerably increased by 25 uM EPA (p<0.01 IC25 and IC50 for oxaliplatin and p<0.01 IC25, p<0.001 IC50 for 5-Fluorouracil), but not SA. We observed that 48 hours of pretreatment with EPA resulted in a statistically significant increase of CK20 expression at the mRNA and protein levels (p<0.05 and p<0.01, respectively) and in a decrease in CD133 mRNA expression (p<0.05) that was consistently and significantly associated with a lowered IC25 and IC50 of the bulk of tumor COLO 320 DM cells to either oxaliplatin or 5-Fluorouracil monotherapy.

In order to study the response of the CD133 (+) CSLCs to the EPA and SA fatty acids, we pretreated magnetically sorted CD133 (+) CSLCs with EPA and SA, or vehicle control. We observed that EPA and SA alone did not impact cellular viability at 48 hours. However, a pretreatment with 25 uM EPA significantly sensitized the CD133 (+) CSLCs specifically to 5-Fluorouracil and not to oxaliplatin (p<0.01 IC25 and p<0.05 IC50).

Various mechanisms have already been identified other than the intrinsic slow proliferation [57] that contribute to CSLCs resistance to conventional anticancer treatments. Among these, the expression of multidrug efflux pumps [58] or alterations in DNA repair system [59].

One of the major obstacles in the therapeutic use of platinum analogues is intrinsic or acquired resistance [60]–[63]. Recently, there have been advances in the understanding of the factors involved in platinum resistance [62]–[64]. Platinum resistance is a multifactor process that may include alteration in drug transport (uptake and efflux), drug detoxification, DNA repair, tolerance to DNA damage, and apoptosis [62], [64].

Uptake and efflux of drugs are regulated by importer and exporter p-glycoproteins (P-gp) that have been classified as ATP-Binding Cassette proteins (ABC-superfamily). Among those the expression of MRP2, one of the multidrug efflux pumps, has been found up-regulated in oxaliplatin-resistant colon cancer cells [65]. EPA and DHA have been shown to reduce gene expression, protein production, and pump activity of MDR1, another ATP-Binding Cassette protein, which is not involved in oxaliplatin resistance [66]. This could explain why EPA pretreatment failed to enhance oxaliplatin sensitivity in CD133 (+) COLO 320 DM CSLCs, but did enhance the sensitivity to 5-Fluorouracil.

Recently, mechanisms of resistance to 5-Fluorouracil in colon cancer have been linked to the activation of the Wnt pathway by the antineoplastic drug [67]. It has been found that 5-Fluorouracil activates the Wnt signaling pathway in CD133 (+) colon CSLCs derived from DLD-1 cells. Interestingly, in Deng et al. following treatments with 5-Fluorouracil the cell proliferation rate of CD133 (+) DLD-1 CSLCs increases compared to the CD133 (−) cells, and the sensitivity of CD133 (+) CSLCs to 5-Fluorouracil decreases [67]. Additionally, the authors found that 5-Flurouracil down-regulated the stem cell marker CD133 expression in colon cancer cell line DLD1 cells. CD133 expressing cells have stronger capacity of clone formation and tumorigenesis [68]. Collectively all these data suggest that treatment of colon cancer cells with 5-Fluorouracil could potentially proliferate and select therapy resistant CD133 (+) colon CSLCs increasing the chances of tumor relapse and metastatic spread.

Our results that treatment of COLO 320 DM cells with 25 uM EPA increased the sensitivity to 5-Fluorouracil in the CD133 (+) cells suggests instead that adjuvant therapy with EPA combined with 5-Fluorouracil could be a viable therapeutic option for colon cancer patients.

Therefore, the effect of 5-Fluorouracil on the activity of Wnt signaling pathway of CD133 (+) colorectal CSLCs, the relation between the activity of the Wnt signaling pathway and drug resistance of stem cells, and the idea of restoring drug sensitivity by blocking the Wnt signaling pathway, all warrant further studies.

Our results showed that EPA treatments decreased the cell number of the bulk of tumor COLO 320 DM without significantly affecting the CD133 (+) CSLCs. Also, we observed that EPA induced down-regulation of CD133 expression and up-regulation of colonic epithelium differentiation markers Cytokeratin 20 and Mucin 2, indicating that n-3 PUFA increased the differentiation status of both bulk of tumor and CSLCs in colon cancer. The pro-differentiating effects of n-3 PUFA could, at least in part, explain the increased sensitivity to 5-Fluorouracil we observed in colon CSLCs following a pretreatment with physiological concentrations of EPA.

In breast cancer others have shown that the down-regulation of CD44 expression, a marker of breast cancer stem cells, was linked to increased sensitivity to doxorubicin treatment [69]. Our results are in agreement with these data as we showed that a reduction in CD133 expression induced by EPA treatments correlated with increased differentiation and sensitivity to chemotherapy in the COLO 320 DM cells.

More studies are needed to elucidate the role of omega-3 fatty acids on the behavior of colon CSLCs. However, understanding the ability of n-3 fatty acids to modulate the plasticity and differentiation status of colon CSLCs may open future avenues in the use of n-3 fatty acids as adjuvant therapy to conventional anticancer drugs *in vitro* and *in vivo*.
